# Femtosecond Laser-Induced Thermal Transport in Silicon with Liquid Cooling Bath

**DOI:** 10.3390/ma12132043

**Published:** 2019-06-26

**Authors:** Zhe Kan, Qinghua Zhu, Haizhou Ren, Mengyan Shen

**Affiliations:** Department of Physics and Applied Physics, and Nanomanufacturing Center, University of Massachusetts Lowell, 1 University Avenue, Lowell, MA 01854, USA

**Keywords:** femtosecond laser irradiation, Silicon, Thermal transport, solidification of capillary wave

## Abstract

Nanostructured regular patterns on silicon surface are made by using femtosecond laser irradiations. This is a novel method that can modify the surface morphology of any large material in an easy, fast, and low-cost way. We irradiate a solid surface with a 400-nm double frequency beam from an 800-nm femtosecond laser, while the solid surface is submerged in a liquid or exposed in air. From the study of multiple-pulses and single-pulse irradiations on silicon, we find the morphologies of nanospikes and capillary waves to follow the same distribution and periodicity. Thermal transport near the solid surface plays an important role in the formation of patterns; a simulation was done to fully understand the mechanism of the pattern formation in single pulse irradiation. The theoretical models include a femtosecond laser pulse function, a two-temperature model (2-T model), and an estimation of interface thermal coupling. The evolution of lattice temperature over time will be calculated first without liquid cooling and then with liquid cooling, which has not been well considered in previous theoretical papers. The lifetime of the capillary wave is found to be longer than the solidification time of the molten silicon only when water cooling is introduced. This allows the capillary wave to be frozen and leaves interesting concentric rings on the silicon surface. The regular nanospikes generated on the silicon surface result from the overlapping capillary waves.

## 1. Introduction

Nanotechnology has contributed significantly to the development of modern manufacturing methods and new energy programs. The performance and efficiency of industrial micro- or nanoelectronic devices have been dramatically improved by the commercialization of various processing technologies in the past few decades. Precise nanoscale processing makes artificial structures or patterns with sizes smaller than 100 nm, which requires the ablation, assembling, and transportation of molecules or even atoms [1]. However, the complexity of the processing, long period of production, availability of substrate, high cost, and low efficiency in the present nanofabrication methods have hindered the development of some potential applications [2]. 

The laser irradiation fabrication method was first discovered from laser mirror damage, where the regular structures were larger than a micrometer [3]. Shen et al. successfully fabricated submicrometer or nanometer regular structures on silicon and other materials by using femtosecond laser irradiations [4,5,6,7,8]. 800-nm or 400-nm femtosecond laser irradiation can form interesting arrays of silicon spikes or pillars. Periodical nanostructures as small as 50 nm have been obtained through similar pulse laser irradiations in various liquids [5,8,9]. These unique structures have many applications, such as field emission, gas sensing, and surface enhancement Raman sensing [8,10,11,12,13]. This method can form submicron structures or nanostructures that are much lower than the laser wavelength. Although it is believed to be easy and fast compared to other nanolithography technologies, the mechanism of structure formation is still not clear.

The laser-irradiation of an ultra-short laser, especially a femtosecond laser, may cause permanent damage to a solid surface. With the irradiation of an ultra-short laser, a flat solid surface absorbs a large amount of energy for a very short amount of time. This can cause ablation on a solid surface, where vaporized species may form nanoparticles or microparticles when the solid is ablated. With the laser ablation method in liquid, nanoparticles with size smaller than 10 nm have been generated from various materials [14,15]. Moreover, it can cause the solid surface to melt, and the re-solidification of the molten surface can leave interesting patterns on the surface. Micro-hole arrays on various metals have been obtained under tightly focused femtosecond laser pulses [13,16]. Patterns of strips, islands, or spikes with very uniform periodicity have been found on silicon surfaces after femtosecond laser irradiations [4,5]. Similar morphologies have been found on metals like iron, steel, titanium, nickel, and coper after laser irradiation in an aqueous environment [6,17,18]. Other than the vaporized or escaped species, the patterns left on sample surface attract our interests because of their regularity in nanoscale and convenience for applications in making devices. Explanations using laser profiles, thermal properties of materials, solidification of capillary waves, photon diffractions, and plasmonic effect have been previously introduced to understand the formation of these patterns [13,16,19,20,21,22,23]. Straight ripples with uniform periodicity have been generated on different material surfaces by using a linearly polarized laser beam, and diffraction mechanism was successfully implemented to explain the formation of these structures [8,24,25]. However, the unique distribution of regular spikes found from 400 nm laser irradiation in water cannot be explained well with photon diffractions [4]. In some previous studies, the size of the patterns becomes much smaller when introducing liquid cooling is introduced to the solid surface [4,5,6]. The formation of these patterns may be associated with not only the heat transfer inside an isotropic lattice but also the heat transport at the solid-liquid interface. However, in previous studies, the liquid cooling system has not been well considered. Therefore, in this paper, the thermal transport of a solid with liquid cooling will be studied to understand the laser-ablation effect on a solid silicon surface.

## 2. Experimental

**Figure 1:**
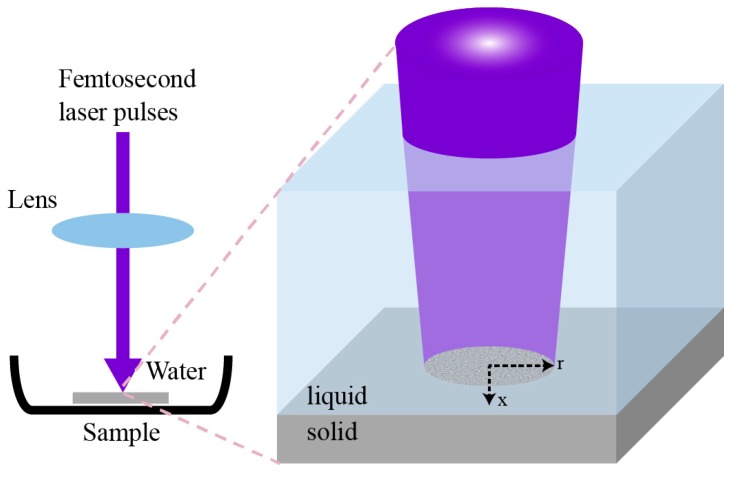
Scheme of femtosecond laser irradiation process.

This experiment was conducted by following the method in Shen et al. [4,5]. The silicon wafer surface was cleaned with hydrofluoric acid and rinsed with distilled water. After drying with compressed nitrogen gas, the substrate was attached to the bottom of a 15-mm deep glass petri dish on a three-axis translation stage. The dish was then filled with distilled water. The silicon surface was irradiated by a 60-mW and 400-nm femtosecond laser generated by a frequency-doubled crystal from a 35-fs 800-nm femtosecond laser. Its pulse energy is 60 µJ, with a spot size of 50 µm, so the corresponding peak power is about 3.4 GW. Compared to the results of 100-fs laser radiation in Shen et al. [4,5], no significant difference in morphology is created by 35-fs laser; the difference in pulse duration does not influence the results of the simulated lattice temperature profiles. The incident laser beam was focused with a 20-cm focal lens and traveled through distilled water perpendicular to the silicon surface. Figure 1 illustrates the interaction between the femtosecond laser and solid substrate. A femtosecond laser is converged to reach the threshold fluence [4,5] to irradiate and damage the solid surface in the Petri dish with cooling water. The right inset of Figure 1 is the schematic diagram of the experimental setup; patterns are generated within the laser spot region. The purple part is the laser beam, the blue part is the liquid cooling system, and the gray part is the solid substrate. The white color at the center of the laser beam indicates the Gaussian profile of the laser. The silicon surface was scanned at a constant speed under the irradiation of consecutive laser pulse at a repetition rate of 1 kHz so that the whole surface can receive uniform exposure to the laser.

**Figure 2:**
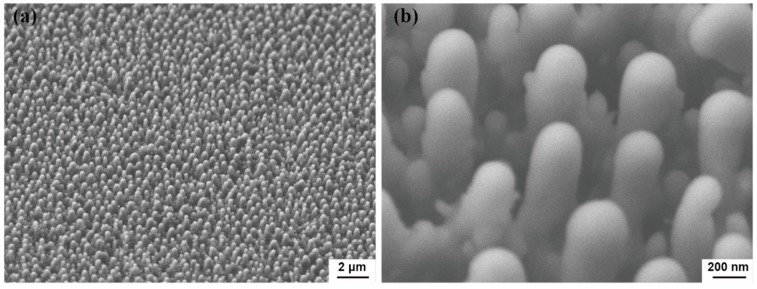
45-degree view FESEM images of silicon foil. (**a**) Region with laser irradiation in water at low-magnification, (**b**) region with laser irradiation in water at high-magnification.

**Figure 3:**
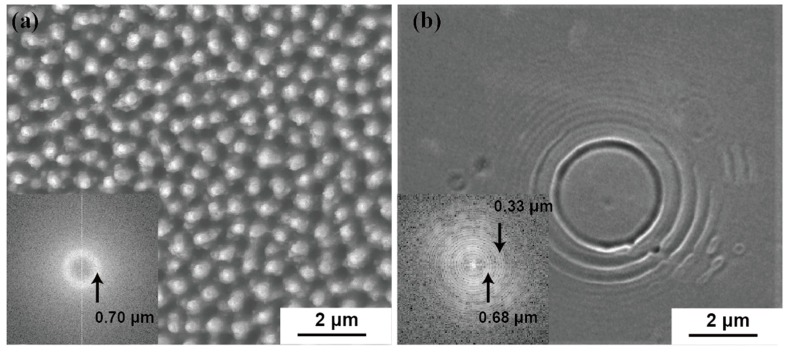
(**a**) Top view FESEM image of silicon foil with laser irradiation in water. (**b**) Top view FESEM image of silicon foil with single pulse irradiation. The insets show Fourier transforms of the images, respectively.

Vertical spikes are generated successfully on the silicon surface after consecutive pulse irradiation. Figure 2 shows SEM image of silicon nano spikes form on silicon foil. The silicon was pre-polished, so there is no image of the smooth surface for comparison. Regular silicon spikes are observed with SEM imaging, shown in Figure 2a. The high magnification SEM image in Figure 2b shows that the size of silicon spikes is about 250 nm in diameter and 600 nm in height. When a silicon surface is irradiated in air instead of water, too much dust is left on the surface. Thus, any potential structure cannot be captured by regular imaging technique. The distribution of the silicon spikes appears random. The repeating spike structures show no preferred direction of growth. A top view image and its Fourier transform image are shown in Figure 3. Single pulse irradiation was also used for studying the mechanism. The corresponding image and its Fourier transform image is shown in Figure 3.

Figure 3a shows a similar SEM image as Figure 2a, but is a top view. Figure 3b shows the SEM image of silicon surface with a single pulse irradiation, and the inset images are their Fourier transforms generated from ImageJ [26,27]. Each point on the bright circle in the Fourier transform of Figure 3a indicates one periodicity along a certain direction found from the bright dot (top of spikes) in the original 2D image. The circular symmetry of the Fourier transform in Figure 3a indicates that the spatial distribution of the spikes is homogeneous in all directions. This is different from the Fourier transforms of straight ripples on silicon generated from 800 nm femtosecond irradiation [5,8], which indicate regularity only along the direction perpendicular to the laser polarization. From the analysis of the Fourier transform in our results, we conclude that the spikes are randomly located on the silicon surface with an average distance of about 700 nm. This is in agreement with the width of the spikes in Figure 2b, measured to be about 250 nm. The separation between spikes is measured to be about 500 nm. Studies of single pulse damage images may provide a hint for the formation of these nano spikes. As shown in Figure 3b, concentric rings were obtained even under one laser pulse irradiation. The Fourier transform of the SEM image shows several bright circles; this indicates that the concentric rings have several different wavelengths. Several inner bright circles are grouped and correspond to a periodicity around 680 nm. These should be from the concentric rings that exhibit large wavelengths in the original 2D image. This periodicity matches the result in Figure 3a, and a noticeable high contrast of the concentric rings indicates that the structures can be very deep. Several outer bright circles are grouped and correspond to a periodicity about 330 nm; they should be from the shallow concentric rings with short wavelengths in the original 2D image. A result of overlapping concentric rings was previously studied and shown in Shen et al. [4]. From our results, we suggest that only deep structures can be retained after the overlapping of many concentric rings, if existing shallow structures elapse easier than deep structures under continuum pulse irradiation. Therefore, the formation of random spikes can be a result of concentric rings. 

Considering that there is no local roughness on polished silicon substrate and the formation of water bubble can take much longer than the pulse duration [28], nothing can diffract the single pulse. One possible cause of the formation of the concentric rings could be the solidification of capillary waves. The size difference or wavelength difference could be due to the solidification time of the capillary wave. The center flat region of the concentric rings on the silicon surface is varied in Shen et al. [4]. They cannot be directly ablated by laser filaments, because each filament carries exactly the same critical power. However, it can be a result of the differences between the lifetime of travelling capillary waves. Some elliptic capillary waves were reported from 790 nm laser irradiation [29]. From our results and Shen et al. [4], we suggest that it is necessary to use a better homogenous beam to obtain circular capillary waves. The penetration depth of 400-nm light in silicon is only 100 nm, which is smaller than the size of the resulting spikes. Meanwhile, that of 800-nm light excesses 10 µm, which is much larger than the size of the resulting spikes [4,5,30,31]. There are different mechanisms in micro/nano-structure formation. The thermal transport after pulse excitation may cause damage or create molten silicon in a region deeper than the laser penetration depth. The mechanism of the temperature evolution and solidification time are covered in our calculation. The overlap of various capillary waves will eventually form a mesh of silicon spikes. Then, the formation of these spikes on a large silicon surface only requires the scan of consecutive pulses by repeating each scan, separated by at least the displacement of the beam waist. Kudryashov et al. have shown a possible surface plasmon resonance near 1 or 2 eV as an origination for the formation of nanopatterns on silicon [23]. The 400-nm light is not tightly focused in the present experiment. Water thickness is typically about 1–2 mm. The consecutive pulse scan showed a damage streak with a width of about 50 µm, which is the approximate laser spot size. This result is shown in Figure S1 in supplementary data. In the experimental setup, in a 2-mm thick water cell, no strong supercontinuum light was generated. The supercontinuum light does not cause damage on silicon surface. The plasmonic resonant wavelength is in the IR because there is a lack of free electrons in silicon. Since we use 400-nm laser radiation, we do not consider the plasmonic effect. 

## 3. Theoretical Method

The formation of capillary wave is studied theoretically through the solidification time of molten silicon induced by a single pulse irradiation. A basic theoretical model, the two-temperature (2-T) model, is used to calculate the thermal transport in solid. The 2-T model is given as two coupled diffusion equations, representing the temperature evolutions of the lattice and electrons, respectively [19,20,21]. The temperature evolution of the silicon lattice shows the process of phase changes over time, and the solidification time will be used for explaining the formation of the patterns left on the surface. A laser pulse function including the laser profiles and its effects on the material will be presented as the driving source of the electron diffusion equation [19,20,21]. 

Most previous works assume a uniform distribution of laser intensive along the transverse direction and neglect the thermal transport along the transverse or radial direction in a solid, a 1D system [19,20,21]. Whereas, most of the laser beams are in a Gaussian profile, and the instantaneous energy distribution along the radial direction in a solid should also be close to a Gaussian. This will lead to thermal transport along the radial direction. Therefore, it is not proper to treat this system as a 1D system, when the laser source exhibits a Gaussian profile. In our calculation, we will implement a 3D calculation of the system, and find out the differences. In addition, some laser irradiation experiments show that regular patterns can be generated only in water cooling systems [4,5,6,7]. A liquid cooling bath at the top surface of the irradiated material will contribute to the cooling of the top surface and then decrease the solidification time of the molten surface. These phenomena and theories drive us to further study the laser ablation effects in a liquid cooling system. Although filamentation can occur when a laser beam travels through an aqueous environment, there is still no explicit way to describe the intensity distribution of each filament [32]. The filamentation may trigger the formation of the circular wave, which is, however, also observed in gas. We believe that the cooling effect of water plays an important role. Here, we used the same intensity distribution to find the differences of time profiles of the temperature of radiated silicon in both air and water. Therefore, the thermal transport at the interface between the solid and the cooling liquid will be only applied at one boundary of the lattice where the laser beam strikes on. It has been studied analytically by characterizing the spring constant of the interaction at the interface [33,34]. After considering these two improvements, some differences in the calculated results are expected, and we will use them to explain our experimental results and predict new phenomena.

As mentioned in Figure 1, the laser beam is Gaussian, having a cylindrical symmetry about its optical axis. Thus, we write the laser pulse function in cylindrical coordinates as shown in the right side diagram in Figure 1. Its time dependent part along the optical axis x is as given [20,21]:

(1) $$Q\left(x,t\right)=\sqrt{\frac{\beta }{\pi }}\;\frac{1-R}{t_{p}\;\delta }I_{0}\;Exp\left[-\frac{x}{\delta }-\beta \;\frac{{\left(t-2t_{p}\right)}^{2}}{t_{p}{}^{2}}\right]$$

where *β* is 4ln2, R is the reflectivity of the solid surface with respect to the laser wavelength, *t_p_* is the pulse duration of the laser, *δ* is the penetration depth of the material, and *I_0_* is the fluence of the laser. Optical axis *x* and time *t* are the basic variables of this 1D laser pulse function.

A radial *r* dependent Gaussian part is multiplied to complete the cylindrical coordinate function. The modified laser pulse function in 3D is then written as:

(2) $$Q\left(x,r,t\right)=\sqrt{\frac{\beta }{\pi }}\;\frac{1-R}{t_{p}\;\delta }I_{0}\;Exp\left[-\frac{x}{\delta }-\beta \;\frac{{\left(t-2t_{p}\right)}^{2}}{t_{p}{}^{2}}\right]\;2Exp\left[-2\;{\left(\frac{r}{\omega }\right)}^{2}\right]$$

where *ω* is the radius of the laser beam at 1/e^2^ in Gaussian profile [35]. According to Shen et al., *ω* = 25 µm is chosen [4,5]. Then the 3D time dependent laser pulse function is completed and it will be used as the driving source of the electron diffusion equation. 

The 2-T model thermal transport is given as two coupled diffusion equations, which represent the temperature evolutions of lattice and electrons, respectively. The electron diffusion equation has *Q* from Equation (2) as the driving source. In a 3-D problem, it is given as [20,21]:

(3) $$C_{e}\frac{\partial T_{e}}{\partial t}=-\nabla \cdot q_{e}-G\left(T_{e}-T_{l}\right)+Q,$$

(4) $$C_{l}\frac{\partial T_{l}}{\partial t}=-\nabla \cdot q_{l}+G\left(T_{e}-T_{l}\right),$$

(5) $$q_{e}=-\lambda_{e}\nabla T_{e},$$

(6) $$q_{l}=-\lambda_{l}\nabla T_{l},$$

where *C* is specific heat, *λ* is thermal conductivity, *T* is temperature, *q* is heat flux vector, *G* is electron-lattice coupling term, and *Q* is laser irradiation function. The subscripts *e* and *l* are assigned to the electrons and lattice, respectively. *T_e_*, *T_l_*, *q_e_*, *q_l_*, and *Q* are functions of *x, r* and *t*. For these equations, *x, r* and *t* are the basic independent variables. 

The system is assumed to be initially in equilibrium with the environment temperature. The neglectable blackbody radiations at the boundaries lead to zero thermal fluxes at the boundaries. Then, the boundaries are written as:

(7) $$T_{e}\left(x,\;r,\;t_{0}\right)=T_{l}\left(x,\;r,\;t_{0}\right)=T_{0},$$

(8) $$q_{e}\left(x_{min},r,t\right)\cdot \hat{x}=q_{e}\left(x_{max},r,t\right)\cdot \hat{x}=q_{l}\left(x_{min},r,t\right)\cdot \hat{x}=q_{l}\left(x_{max},r,t\right)\cdot \hat{x}=q_{e}\left(x,r_{min},t\right)\cdot \hat{r}=q_{e}\left(x,r_{max},t\right)\cdot \hat{r}=q_{l}\left(x,r_{min},t\right)\cdot \hat{r}=q_{l}\left(x,r_{max},t\right)\cdot \hat{r}=0$$

where *T_0_* is the environment temperature at the beginning, *t_0_* is the starting time, $\hat{x}$ and $\hat{r}$ are the unit vectors of the cylindrical coordinate, *x_min_* and *x_amx_* are the boundaries of *x* axis, *r_min_* and *r_max_* are the boundaries of *r* axis.

Up to here, Equations (2)–(8) define the 2-T diffusion equations in a 3-D coordinate with a proper source function and simple boundaries. For the system with liquid cooling, we consider the fluid to be fixed at environment temperature *T_0_*, and the heat flux at this solid-liquid interface equals the temperature difference multiplied by a coefficient *W*. *W* has the SI unit of W/m^2^K. The interface coefficient *W* can be estimated by characterizing the spring constant of the interaction at the interface [33,34]. As it has not been studied under a high-temperature environment [33], we assume that *W* has the value of 10^8^–10^13^ W/m^2^K at high temperatures. Then *x_min_* is chosen at the liquid-solid interface. One boundary in Equation (8) becomes:

(9) $$q_{l}\left(x_{min},r,t\right)\cdot \hat{x}=W\left[T_{0}-T_{l}\left(x_{min},\;r,\;t\right)\right]$$

where the coupling between the solid and liquid only occurs in one lattice boundary equation and there is no electron-liquid coupling considered. Once the silicon temperature reaches above 100 °C, the water on the silicon surface will be vaporized. The water’s latent heat of vaporization causes a large amount of heat transport. Schaffer et al. have shown that laser-induced break down can take 25 ps to form a 0.5 μm radius bubble in water [28]. After the water vaporizes on the hot silicon surface, the boundary condition becomes a solid-gas interface, but the thin layer of vapor in water is not stable and quickly becomes bubbles, which rapidly collapse in water [36]. The water vaporization leads to a larger solid-liquid coupling *W* if we still use the model of Equation (9) for simplicity. However, as we know, there have not been available experimental results to quantitatively describe this cooling process yet, and a reasonable value of *W* cannot be determined at present.

After all, Equations (2)–(9) define the 2-T diffusion equations in a 3D coordinate with the proper source function and boundary conditions. By solving this system, one should be able to obtain *T_e_* and *T_l_* as functions of *x, r*, and *t*. And the evolution of the lattice temperature at the interface *x* = *x_min_* and laser beam edge (also considered as damage edge) is the key to finding the solidification process.

The parameters for silicon irradiated by a 400-nm femtosecond laser are studied and recorded as follows. The reflectivity of the flat surface is found to be about *R* = 0.50 [37]. The penetration depth is found to be about *δ* = 0.10 µm at 400 nm [30,31]. A noticeably large value for 800 nm is found to be about 12 µm. This may correspond to the ripple patterns with a mechanism of photon diffractions [24,38]. The specific heat of the lattice is chosen to be around *C_l_* = 1.0 MJ/m^3^K. We use a constant value because of silicon’s weak temperature dependence at high temperature [39,40]. The thermal conductivity of the lattice is chosen to be *λ_l_* = 130 W/mK for simplicity in this calculation. The specific heat of the electron depends on the electron temperature, and we use a linear relation *C_e_* = *γ T_e_*, where *γ* = 50 J/m^3^K^2^ [41]. We assume electron thermal conductivity in silicon follows the Wiedemann-Franz law, *λ_e_* = *Lσ T_e_*, where Lσ = 24.4 µW/mK^2^ [42]. Following the methodology in Khara et al., the lattice-electron coupling term is calculated to be *G* = 2.8 × 10^17^ W/m^3^K [41]. The solid-liquid coupling coefficient is chosen *W* = 10^8^ W/m^2^K as the description following Equation (9). The environment temperature is chosen to be a constant *T_0_* = 293K. The melting point of silicon is *T_m_* = 1687 K and the boiling point of silicon is *T_b_* = 3538 K.

**Figure 4:**
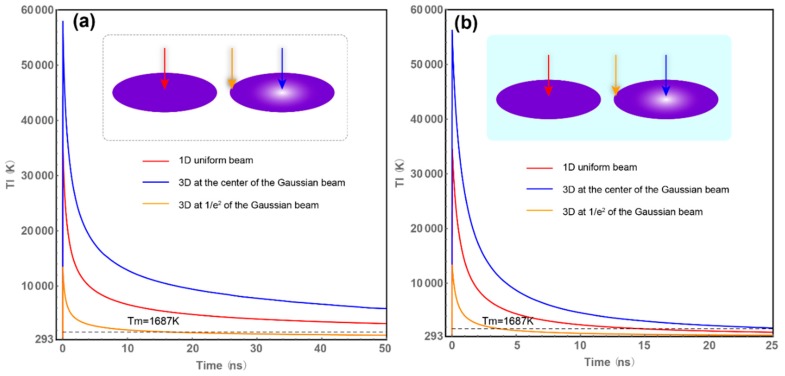
Temperature evolutions of the lattice at the top surface of silicon foil irradiated by 1D uniform beam and 3D Gaussian beam (**a**) without liquid cooling, (**b**) with liquid cooling.

The electron thermal conductivity was studied under low temperature and follows the Wiedemann-Franz law [42]. Danilov et al. showed that electron-ion thermalization can take 1 ps, and after that, lattice heat conductivity dominates the thermal transport [43]. In our calculation, the electron thermal conductivity followed a temperature dependence relation start from Wiedemann-Franz law [42], until it reaches a bandgap temperature of around 10^4^ K [44]. Also, for simplicity, we still use this relation for higher temperature, since our following calculation shows 1.4 W/mK electron thermal conductivity for a maximum temperature 6 × 10^4^ K in Figure 4. The ratio between maximum electron thermal conductivity and lattice thermal conductivity is about 1/100, which is in agreement with the findings in Danilov et al. [43]. The thermal conductivity of silicon below melting temperature has been determined by the steady-state flow technique by Glassbrenner et al [45]. Its value decreases as the temperature increases from room temperature to the melting point of silicon [45]. As the thermal conductivity of silicon varies from 200 W/mK and 10 W/mK in this temperature range, we arbitrarily chose a constant value *λ_l_* for the lattice thermal conductivity for this simulation, which avoids solving high-order differential equations (Equations (4) and (6)). Low thermal conductivity at high temperature may dramatically reduce the heat transport in the lattice, and then cause the high temperature region to have a long temperature decay time. On the other hand, the energy absorbed (e.g., latent heat) during the phase transition in the irradiated silicon may take a certain amount of heat and increase the raising time as well as the dropping time of temperature evolution; this will shrink the high temperature region and decrease temperature decay time. Low thermal conductivity at high temperature and phase transition have different effects on temperature evolution. For simplicity, we used the constant thermal conductivity for the lattice and neglected the energy consumption released during the phase transitions.

## 4. Results and Discussions

We run the simulation in four conditions: 1D without liquid cooling, 1D with liquid cooling, 3D without liquid cooling, and 3D with liquid cooling. Because we are not interested the electron temperatures, only lattice temperatures are shown here. Figure 4a red curve shows the lattice temperature in 1D without liquid cooling. This is the case that has been considered in most of the literature [19,20,21]. Due to the liquid cooling effect, we have to add the liquid-solid interface term. The results are shown in Figure 4b. *T_p_* = 293 K is placed at the bottom of the frames; *T_m_* = 1687 K is the melting point of silicon and is marked by the dashed line. It represents the lifetime of liquid phase silicon. The lattice temperature of silicon raises dramatically and reaches the maximum temperature at about 10 ps, which is much longer than the pulse duration of 35 fs. The maximum temperature of the case with water cooling can be lower than the case without cooling. Water cooling can cool down the lattice temperature slightly, but the surface temperature is still dominated by the electron temperature in this short time interval of pulse duration. After the first pulse, but before the second pulse, there is a time domain of 1 ms for the water to cool down the surface of silicon. From Figure 4b red curve, it takes about 15 ns to drop back to solid phase. On the other hand, the temperature in the case without liquid cooling does not drop back, even after hundreds of ns. Even if we consider the effect of heat transfer between silicon lattice and air gas, or the heat loss from blackbody irradiation, the case without liquid cooling still takes much longer to drop back to solid phase. As the maximum temperature reached 56,000 K, the gas or even plasma phase silicon can be formed. Ablated silicon dust can land on solid surface and remodify its surface morphology. To avoid this, using liquid cooling system can diffuse the silicon dust away from the surface and prevent it from affecting the structured silicon surface. 

Implementation of the 3D calculation may be necessary in order to consider the Gaussian profile of the laser beam. The orange curves in Figure 4 show the temperature evolutions of silicon lattice at the 1/e^2^ Gaussian profile of the beam on top surface. When the laser beam is treated as having a Gaussian profile rather than a uniform profile, a more practical result can be obtained. As the temperatures at the 1/e^2^ Gaussian profile can rise above *T_m_*, a damage region larger than the 1/e^2^ Gaussian profile of the beam should be expected in both two cases. The blue curves in Figure 4 show the temperature evolutions of silicon lattice at the center of the Gaussian profile beam on the top surface. It is not surprising to find that at the center of the Gaussian profile, the temperature of silicon lattice can be larger than the 1D case in red curve. By the definitions in Equations (1) and (2), the intensity at the center of the Gaussian profile of the beam in 3D calculation is twice the case in 1D calculation. But by introducing a 3D system, heat can be dispersed in the 2D surface, which leads the center of the 3D case to be lower in temperature than twice of the case in 1D. The property of a Gaussian-distributed temperature gradient can help the high temperature region disperse the heat. 

The temperature at the center of the Gaussian profile beam can reach 58,000 K in the case without water cooling and 56,000 K in the case with water cooling. It is possible that plasma-phase silicon forms at the center. Recent study on plasma critical temperature of silica has shown a result of 5130 K critical temperature and 0.13 GPa pressure [46]. As silica has a higher melting point and boiling point than silicon, one can predict the critical temperature for silicon to be lower than that of silica. The critical temperature of silicon is chosen at *T_c_* = 5000 K. From our results in Figure 4, at the center of the Gaussian profile beam, plasma phase silicon can be formed; and an explosion will be triggered by the high-pressure plasma. To investigate the actual damage region, the temperature of silicon lattice is mapped in *x*-*r* plane with the coordinates defined in Figure 1. Several time steps were selected to illustrate the evolution of the temperature. 

**Figure 5:**
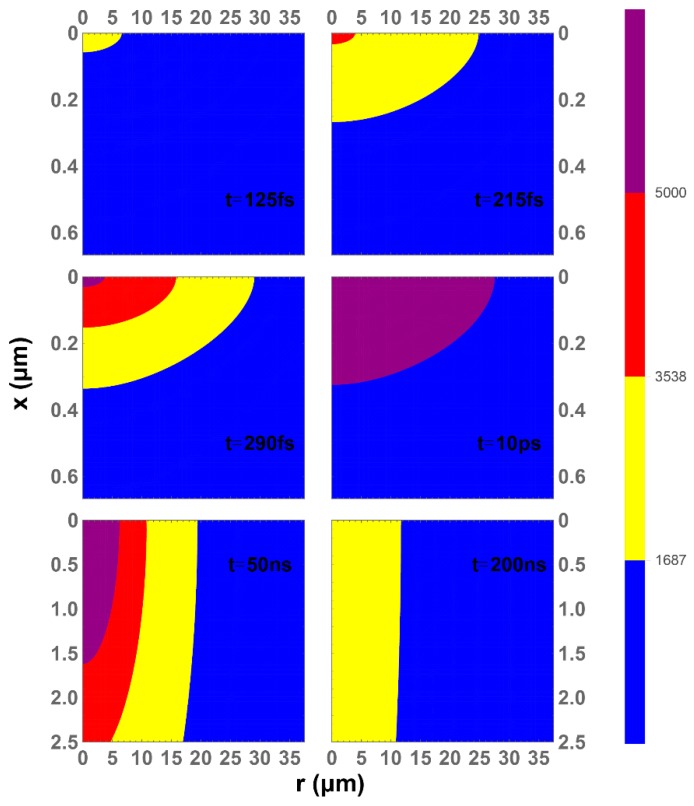
Mapped temperatures of silicon lattice without water cooling at selected time steps.

The temperature evolution of silicon lattice without water cooling is illustrated in Figure 5. x and r are defined in the cylindrical coordinate shown on the right side diagram in Figure 1. The color bar defines the temperature mapping: blue for temperature lower than *T_m_*, yellow for temperature between *T_b_* and *T_m_*, red for temperature between *T_c_* and *T_b_*, and purple for temperature higher than *T_c_*. At 125 fs, the liquid phase region (yellow) begins to appear. It does not disappear even until 200 ns. The gas phase region (red) begins to appear at about 215 fs and the plasma phase region (purple) begins to appear at about 290 fs. These atoms can evaporate and escape to the open space as there is no barrier. At 10 ps, the top surface of the lattice researches its highest temperature and converges with the electron temperature. After this point, thermal diffusion plays a more important role, so a larger plot range in x direction has been used. Multiple phases can be found around 50 ns, and liquid phase and solid phase can remain even until 200 ns. The region of the gas phase (or plasma phase) is smaller than the region of the liquid phase during the entire temperature evolution. Thus, this region evaporates and leaves some damage cavitation, and the rest of the liquid phase region leaves some structures on the silicon. 

**Figure 6:**
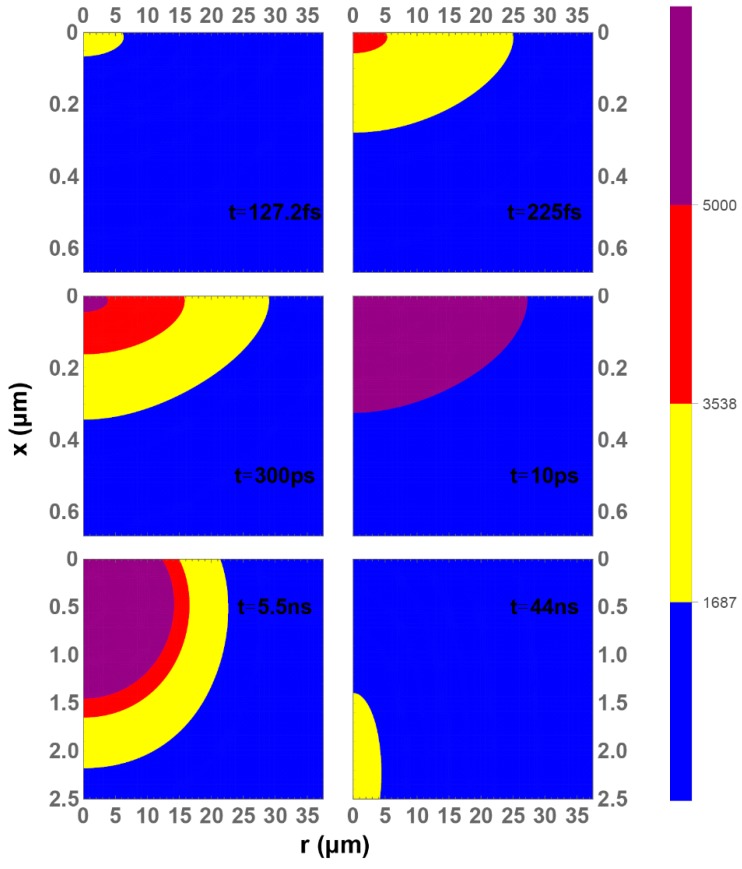
Mapped temperatures of silicon lattice with water cooling at selected time steps.

The temperature evolution of silicon lattice with water cooling is illustrated in Figure 6. At 127 fs, liquid phase region (yellow) begins to appear, similar to the case without water cooling, and it disappears after 44 ns. Because of the water cooling at the top of the silicon surface, the region a few nm below the surface tends to have a higher temperature and give an egg-like high-temperature region, partially enclosed by the low temperature region. The gas phase region (red) begins to appear at about 225 fs and the plasma phase region (purple) begins to appear at about 300 fs. Before the top of the surface can reach its highest temperature at 10 ps, the appearances of the liquid, gas, or plasma phases in this case with water cooling can take slightly longer than the case without water cooling. On the other hand, after 10 ps, the thermal diffusion can be accelerated by the water cooling bath and the liquid phase exhibits a much shorter lifetime than the case without water cooling. Silicon plasma, or gas, move at a very high speed and thus produces a high pressure that can cause an explosion or a shock wave on the surface of the silicon; a capillary wave can be triggered from such a small explosion. The explosion also makes the silicon plasma or gas to escape to the water cooling system and be isolated from the rest of the silicon. It can further allow the rest of the silicon to cool down faster. If the lifetime of the molten silicon is short enough to freeze the capillary wave, the structure such as those depicted in Figure 3 of Shen et al. can be left behind on the surface [4]. 

The lifetime of the liquid phase in this case with water cooling is about 44 ns. This is orders of magnitudes shorter than 200 ns, in the case without water cooling. The lifetime *τ* of a capillary wave with wavelength λ and viscosity *ν* is given as *τ = λ^2^/8π^2^ν*, where *ν* is 2 × 10^−7^ m^2^/s for silicon [5,38,47]. From this calculation, a lifetime of 29 ns is found for the 680-nm capillary wave shown in Figure 3b. As discussed, water cooling can play a major role after the lattice reaches its highest temperature, but the liquid-solid interface coefficient *W* used in the calculation is a conservative estimate. For example, by using *W* = 10^9^ W/m^2^K in our calculation, the lifetime of liquid phase can be shortened to 8 ns. Moreover, when nonlinear losses due to multiphoton absorption and plasma absorption in water are considered [48], the peak power irradiated on silicon submerged in water should be much lower than that of when exposed in air. If a 50% power loss is applied in our calculation, the lifetime of liquid phase can be shortened to 20 ns. The above calculations have shown that plasma phase can be formed during the single pulse irradiation. The lifetimes of the liquid phase can be even shorter if the escaped hot silicon gas or plasma is considered as isolated from the rest of the silicon atoms. As a result, the lifetime of the capillary wave is considered enough for the water cooling case to freeze, but not enough for the case without water cooling. Even if a shock wave is triggered in the case without water cooling, any capillary wave present cannot be solidified due to the long lifetime of molten phase on the surface of silicon.

## 5. Conclusions

Uniform silicon nano spikes are generated with 400 nm femtosecond laser irradiation. To fully understand the formation of these spikes on silicon surface, we have analyzed the surface morphology of the consecutive pulses that irradiate the surface and when a single pulse irradiates the surface. We found that the capillary wave in the molten surface causes the formation of the spikes. The Fourier transform of the images of the spikes matches the Fourier transform of the images of concentric rings. This indicates that the spikes form by overlapping of capillary waves with different wavelengths. Thus, the study of frozen capillary wave is conducted through a laser-induced thermal transport inside the lattice of silicon. 

The 2-T model has been implemented to calculate the temperature evolution of the silicon surface in cases without cooling and with water cooling. In this model, electrons are directly heated by the laser irradiation. The coupling between electron and lattice can further modify the temperature of the lattice with a time delay. Thus, the lattice temperature may be very unstable under electron coupling and cooling system. The case with water cooling can create a small parabolically shaped gas or plasma phase silicon enclosed by the top surface in a few ps. This region can cause a local explosion or shock wave that travels through the large liquid phase region and forms a capillary wave. The lifetime of the capillary wave is found to be longer than the solidification time of the liquid phase silicon, but only when water cooling is introduced. This will allow the capillary wave to be frozen and produce interesting concentric rings on the surface of silicon. Our calculation has explained the experimental results in the work by Shen et al.

By using a time-resolved optical microscopy and pyrometric measurement method, Ionin et al. successfully imaged the silicon surface morphology evolution via laser irradiation in air [49]. It can be slightly modified and applied in our laser irradiation in water, and may provide more data for understanding nanostructure formation. Moreover, in our experiments, we have conducted experiments with compressed gas cooling, water cooling, methanol cooling, and liquid nitrogen cooling systems. Cooling liquids with lower boiling temperatures may exhibit faster solidification, and some cooling systems can prevent the irradiated material from contaminations or chemical reactions (for example, oxidation). Much smaller nanostructures are expected if a sufficient cooling effect is provided.

## Supplementary Materials

The following are available online at https://www.mdpi.com/1996-1944/12/13/2043/s1, Figure S1: Top view FESEM image of silicon foil with continuum beam scan in water.
