# Supplementary material for: Femtosecond Laser-Induced Thermal Transport in Silicon with Liquid Cooling Bath
Source: Materials (Basel). 2019 Jun 26;12(13):2043. doi: 10.3390/ma12132043 (PMC6651069; doi:10.3390/ma12132043)
Supplement: Supplementary file 1 [file materials-12-02043-s001.pdf]

Supplementary Materials: Femtosecond Laser-induced Thermal Transport in Silicon with Liquid Cooling Bath

Zhe Kan *, Qinghua Zhu, Haizhou Ren and Mengyan Shen

Department of Physics and Applied Physics, and Nanomanufacturing Center, University of Massachusetts Lowell, 1 University Avenue, Lowell 01854, MA, USA; Qinghua_Zhu@student.uml.edu (Q.Z.); Haizhou_Ren@student.uml.edu (H.R.); Mengyan_Shen@uml.edu (M.S.)

* Correspondence: zhe_kan@student.uml.edu

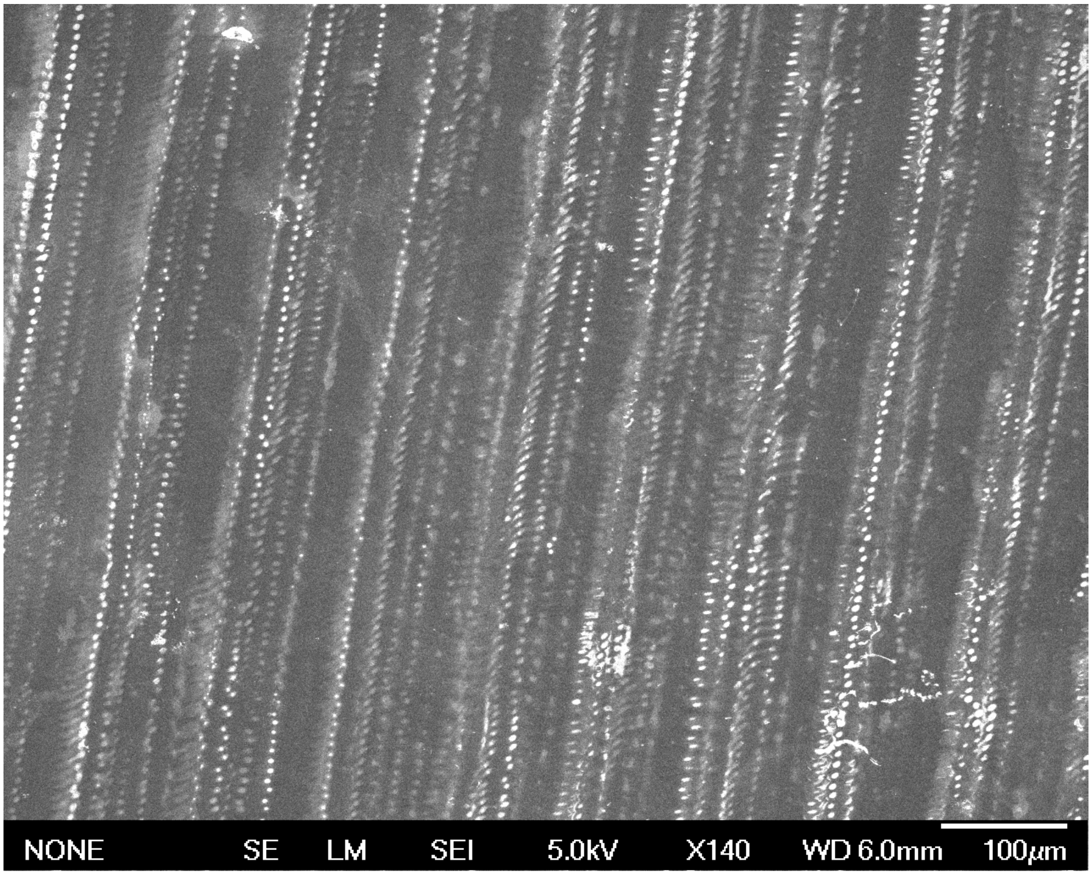

Figure 1. Top view FESEM image of silicon foil with continuum beam scan in water.

We determined the beam waist that strikes the silicon surface from consecutive pulse scan. As described in the experimental section, we only varied the separation displacement between each scan. Then, we found that under 100 μm scan separation, each damage line can be well distinguished. From the SEM image, we conclude that the width of the damaged line can be between 50 and 70 μm. And it corresponds to the beam waist that strikes the silicon surface under the optical setup described in the experimental section.
